# Assessing RGB Color Reliability via Simultaneous Comparison with Hyperspectral Data on Pantone^®^ Fabrics

**DOI:** 10.3390/jimaging12030116

**Published:** 2026-03-10

**Authors:** Cindy Lorena Gómez-Heredia, Jose David Ardila-Useda, Andrés Felipe Cerón-Molina, Jhonny Osorio-Gallego, Jorge Andrés Ramírez-Rincón

**Affiliations:** 1 Grupo de Películas Delgadas y Nanofotónica, Departamento de Física, Facultad de Ciencias, Pontificia Universidad Javeriana, Bogotá 110231, Colombia; 2 Grupo de Investigación en Ciencias y Educación (ICE), Facultad de ingeniería, Universidad de América, Bogotá 110211, Colombia; jose.ardila@uamerica.edu.co (J.D.A.-U.); andres.ceron@uamerica.edu.co (A.F.C.-M.); j.osorio@uamerica.edu.co (J.O.-G.)

**Keywords:** hyperspectral imaging, sRGB-REC2020 images, CIE-L*a*b* color space, relative color differences

## Abstract

Accurate color property measurements are critical for advancing artificial vision in real-time industrial applications. RGB imaging remains highly applicable and widely used due to its practicality, accessibility, and high spatial resolution. However, significant uncertainties in extracting chromatic information highlight the need to define when conventional digital images can reliably provide accurate color data. This work simultaneously compares six chromatic properties across 700 Pantone^®^ TCX fabric samples, using optical data acquired simultaneously from both hyperspectral (HSI) and digital (RGB) cameras. The results indicate that the accurate interpretation of optical information from RGB (sRGB and REC2020) images is significantly influenced by lightness ($L^{*}$) values. Samples with bright and unsaturated colors ($L^{*}>$ 50) reach ratio-to-performance-deviation (RPD) values above 2.5 for four properties ($L^{*},\;a^{*},\;b^{*}$ $h_{ab}$), indicating a good correlation between HSI and RGB information. Absolute color difference comparisons (${∆E}_{a}$) between HSI and RGB images yield values exceeding 5.5 units for red-yellow-green samples and up to 9.0 units for blue and purple tones. In contrast, relative color differences (${∆E}_{r}$) comparisons show a significant decrease, with values falling below 3.0 for all lightness values, indicating the practical equivalence of both methodologies according to the Two One-Sided Test (TOST) statistical analysis. These results confirm that RGB imagery achieves reliable color consistency when evaluated against a practical reference.

## 1. Introduction

Color appearance is a primary sensory attribute commonly linked to the quality, purity, and chemical composition of an object. Rather than representing intrinsic properties of a sample, color reflects the visual response arising from its optical characteristics interacting with an illumination source [1,2,3,4]. Important processes in industries such as automotive, textile, food, pharmaceutical, cosmetic, and packaging require thorough color inspection for tasks such as painting matching, fabric dyeing, garment color consistency, ensuring food and beverage quality, medication identification and quality, hair dye formulation, makeup consistency, and packaging design [5,6]. These processes are crucial for enhancing product appeal, preserving brand consistency, and ensuring consumer satisfaction. However, color evaluation has traditionally relied on visual inspection by trained personnel, whose assessments may vary due to factors such as lighting conditions, individual perceptual differences, and visual fatigue—introducing inherent ambiguities and inconsistencies [7,8].

In recent decades, advances in artificial and computer vision driven by image/video processing technologies and sophisticated algorithms, have enabled automated identification, classification, and real-time assessment in industries such as pharmaceuticals [9,10], food [11,12], and textiles [13]. For agriculture, Rodríguez-Pulido et al. integrated color and NIR spectral data for simultaneous pixel- and object-level evaluation of grape and seed samples. Similarly, machine vision applied to strawberries combined shape, size, and color analysis through line drawing, K-means clustering, and Multi-attribute Decision Making Theory, achieving color accuracy of 88.8%, shape classification above 90%, and size error detection of 5% within three seconds [14]. In textiles, Çelik et al. designed a fabric inspection system using a feedforward neural network with accuracies of 93.4% (defect detection) and 96.3% (classification) [15]. Later, Dlamini et al. developed a system combining image acquisition hardware with YOLOv4-based processing, achieving 95.3% precision at 34 fps [16].

Recently, hyperspectral imaging (HSI) has emerged as a cutting-edge tool in production lines, medical diagnostics, precision agriculture [17,18,19], and packaging [20], replacing traditional methods for evaluating sensory/color and physicochemical properties. Unlike point spectrometers or colorimeters, HSI simultaneously captures high spatial (millions of pixels) and spectral (numerous bands) information, proving valuable both industrially and scientifically [21]. However, its large scale or even laboratory use is limited by high equipment costs and the expertise required for acquisition, processing, and analysis [22,23,24]. Additionally, both spectroscopy and HSI generate vast datasets across full wavelength ranges that require careful preprocessing before modeling [25,26]. In contrast, RGB imaging, using simple digital cameras with color filters to capture three spectral bands, remains preferable where analysis areas are limited, high spatial resolution and short shutter times are needed, or conditions challenge instrument durability [27].

Although RGB data provides spectral information from only three broad bands, accurate classification can be achieved by leveraging morphological and spatial features [28]. A review in Ref. [29] compiles studies on spectroscopy, colorimeters, and hyperspectral imaging combined with Machine Learning for rapid and non-destructive crop weed discrimination. Conventional spectroscopy delivers extensive VIS/IR data strongly correlated with plant species, though optimal ranges depend on crop and weed characteristics. Hyperspectral imaging shows high potential for real-time identification but requires optimized input variable selection. In contrast, RGB imaging, despite its limited spectral resolution, has proven highly effective for plant classification. Recent efforts focus on training Neural Networks to expand RGB data into more than 50 optical bands, with promising results, though their applicability remains dependent on specific training, testing, and experimental datasets [30,31,32].

In the visible range (380–780 nm), RGB imaging enables color contrast generation through channel intensity changes, facilitating chromatic variation estimation, mainly via lightness, with applications in agriculture and healthcare [33,34,35,36]. This approach remains widely used, even with higher resolution images, as it is simple, reliable, and requires no advanced processing skills [37,38]. However, precise and absolute color calculations rely on spectrometers or hyperspectral cameras, which accurately determine CIE-$L^{*}a^{*}b^{*}$ coordinates [39]. For instance, Lasarte et al. enhanced RGB-CCD measurements with chromatic filters, achieving over 96% color reproduction accuracy and improved appearance parameter predictions using Hunt’94 or CIECAM02 models compared to CIE-$L^{*}a^{*}b^{*}$ [40]. Similarly, hyperspectral imaging outperformed RGB (95.83% vs. 66.2% accuracy) in discriminating apple maturity under different storage conditions [41]. Nonetheless, RGB imaging struggles to match HSI precision due to the non-uniformity of the visible spectrum and color representation variability to each RGB model (sRGB, Adobe RGB, ProPhoto RGB, REC2020, etc.) [42,43]. Consequently, defining the conditions under which RGB images can reliably reproduce chromatic properties is essential to expand machine vision applications in color-sensitive fields.

This work presents a comprehensive and simultaneous comparison of six chromatic properties—$L^{*}$ (lightness), $a^{*}{,\;b}^{*}$, $C_{ab}^{*}$ (chroma), $h_{ab}$(hue), and S (saturation)—across 700 Pantone^®^ TCX fabric samples, using optical information captured from both hyperspectral and digital images. The experimental setup involved capturing HSI and RGB images of the fabric samples under uniform illumination conditions. A custom Python algorithm was developed to automatically normalize the images relative to a white reference, isolate each sample, extract its optical data, convert it to the CIE-L*a*b* color space, and conduct a detailed comparison between the two imaging modalities. This methodological framework enabled a robust and scalable evaluation of chromatic fidelity across a large and diverse dataset. The results offer valuable insights into the conditions under which RGB images in the sRGB and REC2020 representations (different color gamut) can serve as a reliable alternative to hyperspectral imaging for accurate color characterization, highlighting the practical potential of RGB systems in applications where hyperspectral imaging may not be feasible.

## 2. Materials and Methods

### 2.1. Experimental Setup

**Figure 1:**
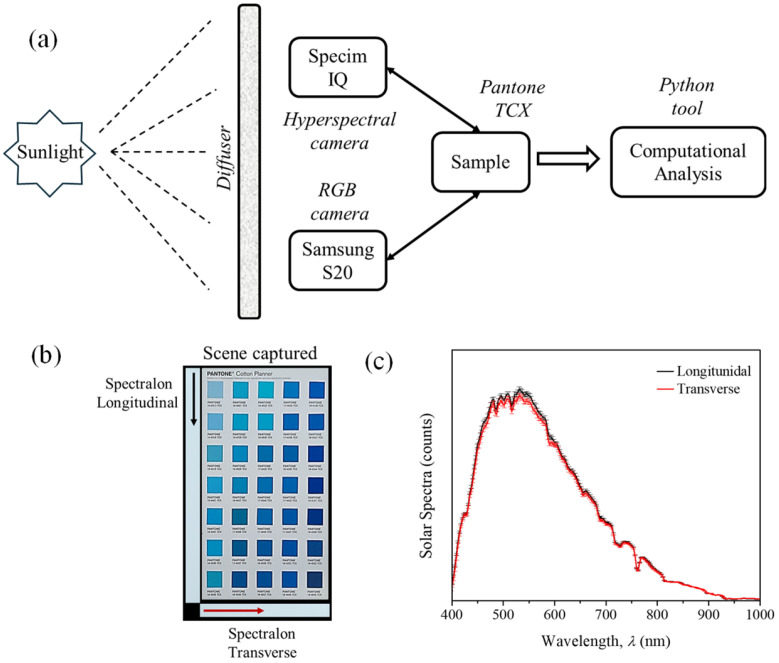
(**a**) Experimental setup used for the acquisition and analysis of hyperspectral and RGB images. (**b**) Real captured scene illustrating the arrangement of 35 fabrics and two Spectralon references in each image, used to verify the stability and homogeneity of sunlight. (**c**) Average solar spectra obtained from the longitudinal (black) and transverse (red) Spectralon references. The error bars indicate the intensity variations across the analyzed area.

HSI and RGB images were simultaneously acquired under natural sun lighting on a clear day around noon with oblique incidence to ensure uniform and stable illumination over the area of analysis. The experimental setup included two cameras (HSI and RGB) aligned frontally at a fixed height of 45 cm above the samples. A diffuser surface was employed to homogenize the illumination, effectively minimizing shadows and saturation artifacts (see Figure 1a). As white reference, two Spectralon targets were placed along the longitudinal and transverse directions to verify the stability and homogeneity of spectral power distribution (SPD) in each image (Figure 1b), thereby reducing errors in both HSI and RGB data during capturing (30 s for HSI and 1/60 s for RGB) [44]. The SPD variations measured in each image were below 3% (Figure 1c) which was considered sufficiently homogenous for the 17 × 27 cm^2^ scene analyzed in this work. For the hyperspectral camera, normalization was performed automatically using the “simultaneous reference mode”, whereas for the RGB images, it was applied during the computational processing stage. The hyperspectral camera was operated with an integration time of 3 ms. RGB images were acquired in PRO mode, with an ISO setting of 6400 and autofocus enable.

#### 2.1.1. Hyperspectral and Digital Cameras

Hyperspectral images were acquired using the Specim IQ handheld camera, featuring a resolution of 512 × 512 pixels and 204 spectral bands within the 397 to 1003 nm range (±2.97 nm). The camera utilizes an NVIDIA Tegra K1 processor (SPECIM, Spectral Imaging Ltd, Oulu, Finland) with CMOS technology and a 5 Mpx viewfinder, employing an optical motion engine in a pushbroom configuration for image generation [45]. Conventional digital RGB images were captured using a Samsung S20 mobile phone with a 16-megapixel CMOS camera (3024 × 4032 resolution). Images were saved in RAW format to retain uncompressed and unprocessed data to be subsequently displayed in sRGB and REC2020 formats [46], which cover the 35% and 72% of visible color space, respectively [43,47].

#### 2.1.2. Pantone TCX

To compare the color properties derived from HSI and RGB images, 700 fabric samples from the Pantone^®^ Fashion, Home + Interiors Cotton Planner—an industry-standard reference in the textile sector—were used. Twenty pages, each containing 35 samples, were selected to represent a broad range of hues across the chromatic circle [48], as shown in Figure S1 [49,50,51] of the Supplementary Materials. The optical data from the TCX fabrics were converted to the CIE-L*a*b* color space using a standard 2° observer and D65 illuminant.

### 2.2. Computational Process

**Figure 2:**
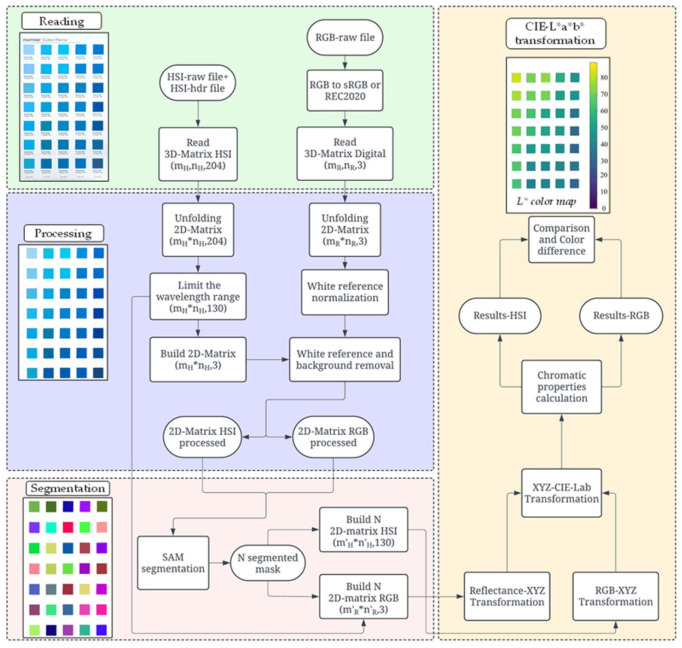
Flowchart of the Python tool (v. 3.12) developed for reading, processing, segmenting, and transforming hyperspectral and RGB images into the CIE-L*a*b* color space. The images illustrate the step-by-step transformation applied to data as part of the computational algorithm.

The optical information contained in both the hyperspectral fingerprint and RGB values must be analyzed at the pixel level. To facilitate this process, a Python-based tool was developed to automatically normalize, read, process, segment, and compute the chromatic properties of each sample. Figure 2 presents a flowchart illustrating the algorithmic steps used to convert the images into the CIE-L*a*b* color space.

#### 2.2.1. Image Reading

The algorithm employs two parallel processing lines that merge during the comparison of the results. The initial step involves reading the images using the libraries Spectral (HSI) and OpenCV (RGB). The images are stored as 3D hypercubes with dimensions $\left[m_{H},\;n_{H},\;204\right]$ for HSI and $\left[m_{R},\;n_{R},\;3\right]$ for RGB, corresponding to the number of rows (m), columns (n) and spectral bands contained [52].

#### 2.2.2. Processing

The hypercubes are “unfolded” using the Numpy library into 2D arrays of dimension $\left[m_{H}*n_{H},\;204\right]$ and $\left[m_{R}*n_{R},\;3\right]$, facilitating the matrix operations for transformation into CIE-L*a*b* color space. The hyperspectral data is limited to the 400–780 nm range (130 bands), within which the color properties are calculated [53,54,55]. The standard deviation of the optical data across pixels is used to identify and exclude the white reference and background from the analysis [56]. For RGB images, the white reference coordinates are similarly employed to normalize and scale the data from 0 to 1, a necessary step for the application of the XYZ conversion matrices [57,58].

#### 2.2.3. Segmentation

The segmentation of the 35 samples in each image was performed using the Segment Anything Model (SAM) via the Segment Anything library [59]. A total of 700 samples were evaluated; however, they were confined within 20 structured images, each containing 35 objects arranged in a fixed grid (columns 1–5 and rows 1–7, see Figure 2). This deterministic layout enabled systematic indexing and direct correspondence between RGB and HSI images. In this controlled setup, segmentation accuracy was visually verified, and no ambiguities such as partial boundaries, multiple segment assignments, or confusion with the background were observed, which implies confidence in the segmentation results. For HSI images, a dimensional reduction is required using the bands of red (600 nm), green (550 nm), and blue (452 nm), preserving the spectral fingerprint of each sample. This process generates N matrices (masks) associated with the number of identified objects. In this study, 2100 matrices were obtained (35 fabrics × 20 photos × 3 types: HSI, sRGB, REC2020) with dimensions $\left[{m\prime }_{H}*{n\prime }_{H},\;130\right]$ for HSI and $\left[{m\prime }_{R}*{n\prime }_{R},\;3\right]$ for RGB. Note $m^{\prime }*\;n^{\prime }\ll m*n$, as each object occupies a small fraction of the total image area.

#### 2.2.4. CIE-L*a*b* Transformation

The matrices generated are systematically numbered and organized to facilitate comparisons. This data is then transformed into coordinates XYZ by matrix operations using Equation (S5) (HSI) [60,61,62,63,64,65,66], Equation (S9) (RGB) [47,67,68,69] and therefore calculated the L*a*b* values using Equations (S6)–(S8). The chromatic information reported in the results section corresponds to the average over 550 pixels for HSI and 23 k pixels for RGB, obtaining surface uniformities higher to 95% for all cases, which indicates the homogeneity of samples analyzed. The algorithm then separates and organizes the information into four quadrants (Q) based on their position in the CIE-L*a*b* color space, mainly using the Hue ($h_{ab}$) parameter: Q1 (0–90°), Q2 (90–180°), Q3 (180–270°), and Q4 (270–360°), as illustrated in the chromatic circle (Figure S1) of the Supplementary Materials.

#### 2.2.5. HSI–RGB Color Comparison

Color differences have been reported as a fundamental parameter in both academic research and industrial applications of chromatic inspection. It is particularly useful for establishing perceptual similarities through direct comparison of a sample’s color properties with respect to a reference, as well as for evaluating the equivalence between different colorimetric instruments [70,71]. Several mathematical definitions for the color difference calculation are currently used [72], which mainly differ in the number of parameters involved. The simplest definition employs the Euclidean distance between two points in CIE-L*a*b* space (Equation (S4)). However, due to the strong dependence of hue and lightness with the visual perception of color, alternative definitions such as CIE-2000 (∆E) have been deemed for assessing these differences [73]. Detailed descriptions of each parameter involved in Equation (1) are available in Ref. [74].

(1) ∆E=ΔL′KLSL2+ΔC′KCSC2+ΔH′KHSH2+RTΔC′KCSCΔH′KHSH12.

In our work, the CIE-2000 color difference was used to analyze the equivalence of the chromatic information obtained from HSI and RGB images in both representations (sRGB and REC2020) through two comparative metrics [75,76]:

1. Absolute color difference (${∆E}_{a}$): Calculated by directly comparing the color properties obtained for each sample from HSI and RGB images. This quantifies the accuracy of RGB data to reproduce a color perception similar to that of HSI.
2. Relative color difference (${∆E}_{r}$): Calculated by selecting one sample as a reference within each imagen and computing the color differences in the remaining 34 samples. This process was performed automatically with the Phyton tool, using as references the first sample in the upper-left corner on HSI and RGB images independently (see Figure 2). The results were subsequently compared to quantify the reliability of RGB data in reproducing color differences between samples within the same image.

Although a unique threshold for perceptual similarity in a color dataset has not been established, recent academic and industrial studies indicate that color differences below 3 units (∆E < 3) are generally sufficient to consider two or more processes chromatically equivalent [77,78,79,80].

## 3. Results

### 3.1. Optical Characterization

**Figure 3:**
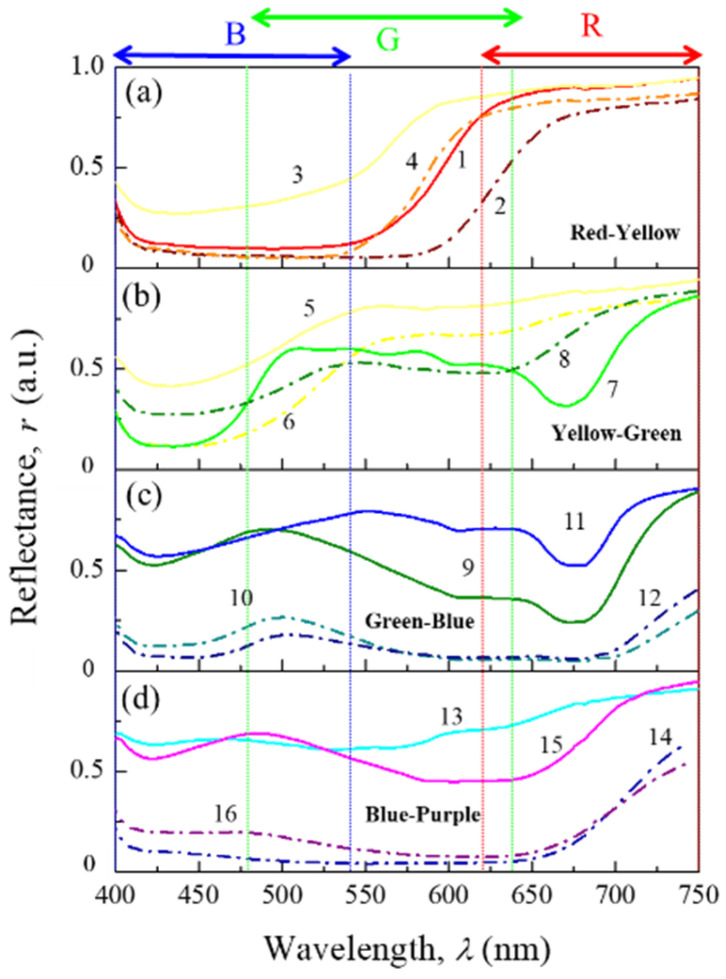
Reflectance spectrum of 16 representative Pantone^®^ fabrics samples (TCX) in the visible range (400–750 nm) separated by light (solid line) and dark (dash line) colors in the chromatic regions of (**a**) red-yellow, (**b**) yellow-green, (**c**) green-blue and (**d**) blue-purple. The detection bands R (red), G (green) and B (blue) are included (vertical dash lines) as reference to correlate the spectral fingerprint with the corresponding RGB data.

Figure 3 shows the reflectance spectra in the visible range of 16 Pantone^®^ fabric samples whose chromatic properties are representative of CIE-L*a*b* color space. Moreover, the typical spectral sensibility of conventional cameras for R (red), G (green) and B (blue) bands are included (vertical dashed lines). For the red-yellow group (Figure 3a), a notably higher reflectance is observed in the red region (>600 nm) compared to the blue one (<500 nm); therefore, their chromatic properties are expected to be in the first quadrant (Q1) of chromatic circle. In the case of samples 5 to 8 (Figure 3b), although the reflectance is also high in the red region, there is also a significant increase (>50%) of the signal in the green band, therefore these colors will be a combination of yellow and green tones (Q2). The spectra of samples 9 and 12 (Figure 3c—Q3), as well of 13 and 16 (Figure 3d—Q4) present similar characteristics in the blue region but mainly differ in the signal intensity in the green and bands. Likewise, the comparison between samples 3–4 and 5–6, exhibits comparable spectral signatures with different intensities, resulting in lighter or darker colors associated with increases or decreases in reflectance signals. This latter can be observed in curves 10–12 and 14–16 where the colors tend to be darker (low lightness) because a wide amount of visible light is absorbed. These findings are further supported by the color representation of the 16 samples in both the sRGB and REC2020, as shown in Table S1 of the Supplementary Materials.

### 3.2. Color Properties from Hyperspectral Images (HSI)

The color properties of the 700 samples analyzed in this study were derived from the examination of HSI and RGB images using the computational tool and methodology outlined in Section 2.2. The chromatic information obtained from the HSI is summarized through the statistical analysis presented in Table 1. It is worth noting that each quadrant includes samples with lightness (L*) values ranging from 20 to 95, encompassing both light and dark color appearances. Similarly, the a and b coordinates, along with the derived chroma ($C_{ab}^{*}$) and saturation (*S*) values, span from very low intensities (<5) to values approaching the limits of the visible color space (>80). The hue ($h_{ab}$) values also cover the entire chromatic circle, ranging from 0° in Q1 to 360° in Q4. Furthermore, the HSI-derived chromatic properties were compared with the reference values provided by the manufacturer for all 700 samples, as reported online [81]. Differences ($\bar{∆P}$) below 3.0 were observed for each property across all quadrants, highlighting the reliability of the HSI data and, subsequently, the accuracy of the color properties calculated from the hyperspectral images. This comprehensive coverage underscores the dataset’s robust statistical significance in characterizing chromatic properties within the CIE-L*a*b* color space.

The mean and median values of $L^{*}$ and $C_{ab}^{*}$ across all groups are below 70 and 35, respectively, indicating a general tendency toward darker colors. This may hinder accurate interpretation of optical information from RGB images, particularly in Q3, due to (i) the limited range of colors represented in each color space and (ii) the expansion of the visible color gamut as $L^{*}$ decreases [82,83].

### 3.3. Comparative Analysis of Color Properties from HSI and RGB Images

**Figure 4:**
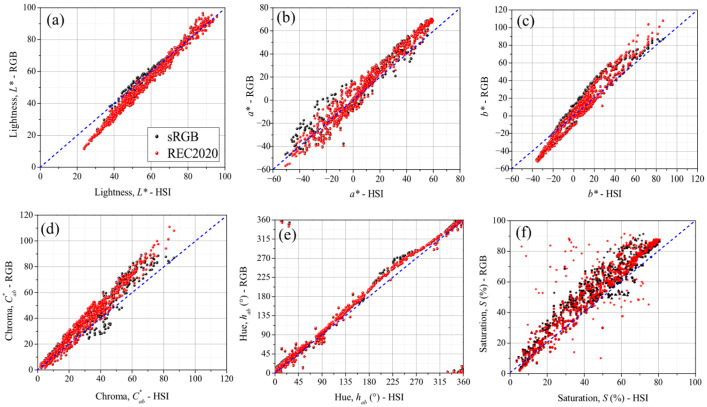
Scatter plots of the chromatic properties (**a**) lightness $L^{*}$, (**b**) $a^{*}$, (**c**) $b^{*}$, (**d**) chroma, (**e**) hue and (**f**) saturation, obtained from hyperspectral and RGB images in the representations sRGB (black) and REC2020 (red). The dash blue line is included in each case as a reference to observing the region of better agreement between HSI and RGB data. The icons in saturation plot have been separated by quadrant: squares (Q1), circles (Q2), triangles (Q3) and stars (Q4).

Figure 4 presents scatter plots for the L*, a*, b*, $C_{ab}^{*}$, $h_{ab}$, and  S color properties, comparing the values obtained from RGB images in both sRGB and REC2020 color spaces with reference values generated from the HSI data. A dashed blue line in each plot represents the perfect correlation between HSI and RGB. The lightness (in Figure 4a) shows a better HSI-RGB agreement for values higher than 60 units. For lower values, L* exhibits an underestimation related to the RGB camera’s capability to capture and interpret optical information in conditions of low reflectance/transmittance, which in turn affects the calculation of the Y stimulus. In consequence, the reproduction of a* and b* is seriously compromised by the most intense colors, where a noticeable increase in dispersion is observed for values a* < 0 and b* > 0, specifically for chromatic coordinates located in Q2 and Q3 (Figure 4b,c). Eventually, the chroma (Figure 4d) also exhibits an overestimation for $C_{ab}^{*}$ > 40 in colors with relative high purity. The best HSI-RGB relationship is found in the region C < 30, where a* and b* tend to zero with b* close to white reference.

On the other hand, the hue demonstrates excellent reproducibility between optical and digital information (Figure 4e). Therefore, reliable chromatic contrasts for the identification and classification of colors within a set of samples can be achieved. The regions of major dispersion of $h_{ab}$ are observed in both cases when the quadrant changes due to a decrease to zero and when a* and/or b* change signs. This significantly affects the tangent function used to calculate $h_{ab}$, producing different tones than expected and even changing the color space quadrant. The best reproduction occurs in Q2, where the samples have the highest L* values and the lowest *S* values, resulting in a better HSI-RGB correlation. Conversely, in the range between 180° and 270° (Q3), where a* and b* values—and consequently $h_{ab}$—exhibit the greatest uncertainty, a poor correlation is observed.

Saturation is the color property with the lowest HSI-RGB reproducibility (Figure 4f) and shows the highest difference between sRGB and REC2020 representations. In the first case, values of up to 90% associated with high purity color samples have been obtained. However, this prediction is incorrect when considering the reference dataset information in Table 1, where the maximum value of *S* obtained is lower than 80%. “When the color saturation in the digital representation approaches 100%, at least one of the RGB channels tends toward zero. As a result, the xy chromaticity coordinates shift toward the boundary of the color space (see Figure S2 in the Supplementary Materials), thereby limiting the accuracy of color representation. The greatest disparities in saturation are found in Q4 (corresponding to the line of purples in the visible color space), where the colors are primarily combinations of the red and blue channels—conditions that, as previously mentioned, hinder accurate digital representation.

It is worth noting that although REC2020 encompasses approximately 72% of the visible color space and it is expected to provide predictions closer to those from HSI, differences in saturation (S) of up to 30 units are still observed. These discrepancies can be attributed to the optical information obtained from RGB camera, which is strongly conditioned by the spectral response of the Bayer array filters (RGGB) in the cellphone, thereby limiting the information captured in each channel [84,85]. Although the RGB data extracted from RAW files yields sRGB and REC2020 values that appear quite different (Table S1), the corresponding conversion matrices sRGB → XYZ and REC2020 → XYZ (Equation (S9)) produce color data with similar chromaticity coordinates (xy) i.e., similar perceptual colors as shown in Figure 4. This outcome suggests that the rescaling process from RAW to REC2020 is essentially artificial and does not expand the effective capacity (gamut) of the sensor to generate more accurate images. Consequently, from the outset, the range of color information that can reproduce, particularly in saturated and pure colors, is inherently limited [86,87].

## 4. Discussion

The scatter plots presented in Figure 4 offer a powerful tool to provide a comprehensive view of when chromatic properties can be successfully reproduced through RGB information, as well as the specific value ranges where this reproducibility holds true. However, the proximity of color properties in both representations (sRGB and REC2020), considering the differences in color space, can lead to ambiguous interpretations. To evaluate the accuracy of the proposed method in reproducing HSI chromatic information through the sRGB and REC2020 representations, the ratio-to-performance deviation (RPD) is calculated. This metric quantifies the deviation between predicted (RGB) and actual (HSI) values by comparing the Standard Deviation (SD) of actual data with the Root Mean Square Error (RMSE) of predicted values. Based on the RPD results, the relationship between dataset can be classified as Excellent (RPD > 3), Good (2.5 < RPD < 3.0), Approximate (2.0 < RPD < 2.5) or Unsatisfactory (RPD < 2.0) [88,89]. Table 2 presents the evaluation of how accurately each color property is represented across different quadrants and lightness intervals within each RGB representation.

Lightness is the parameter that gives the better reliability, especially for quadrants Q1 and Q2, where the highest values of $L^{*}$ are found in the dataset. In contrast, dark and highly saturated colors exhibit RPD values below 1.0, highlighting the strong dependence of RGB information on sample lightness. The uncertainty in $a^{*}$ and $b^{*}$ values become more pronounced for $L^{*}\leq$ 50, and this effect is amplified in the chroma ($C_{ab}^{*}$) parameter due to their quadratic relationship (Equation (S1)). This lightness dependence also affects $a^{*}$ and $b^{*}$, with more accurate RGB-based predictions observed when $L^{*}$ is greater than 50. Generally, for bright and unsaturated colors ($L^{*}>$ 50), sRGB and REC2020 predictions show a remarkable agreement related to HSI, with RDPs > 2.5 for at least 4 of the 6 chromatic properties assessed (Classified as Good and Excellent). This consistency between both RGB representations indicates that the matrices defined for each CIE-XYZ transformation (Equation (S9)) enable the recovery of similar color data from any RGB image.

### Color Differences Between HSI and RGB Images

**Figure 5:**
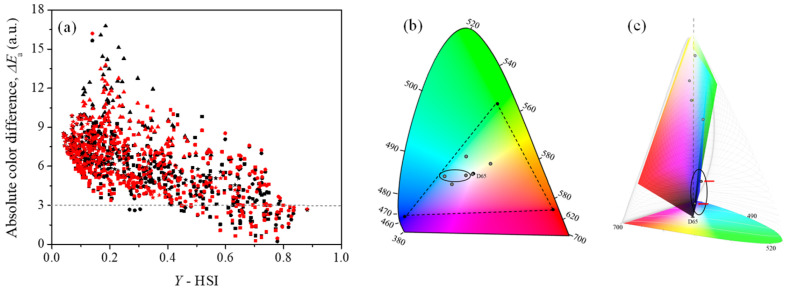
(**a**) Scatter plot of the Y-stimulus obtained from HSI images and absolute color difference (∆*E*) between HSI and RGB data, for the 700 Pantone^®^ fabric samples (TCX) in sRGB (black) and REC2020 (red) representations. The icons have been separated by quadrants: squares (Q1), circles (Q2), triangles (Q3) and stars (Q4). The coordinates of six samples with different Y-stimulus values are displayed in (**b**) CIE-xy chromaticity and (**c**) CIE-xyY diagrams, which illustrate the spectral combinations that can produce the colors perceivable by human eye. Circle indicates the samples outside the sRGB volume.

Considering that the number of perceivable colors in the visible space decreases as the Y-stimulus (luminance) increases [82,83], the proportion of colors encompassed by the RGB representations become comparatively higher. Therefore, it is expected that as the chromatic properties of a sample approach to the reference white point (*Y* → 1), its digital reproduction will more closely align with the HSI image information. Figure 5a presents the scatter plot of the Y-stimulus, derived from HSI data, for each sample alongside the corresponding HSI-RGB absolute color difference defined in Section 2.2.5 (${∆E}_{a}$). A general trend indicates that ${∆E}_{a}$ decreases as *Y* increases. For *Y* > 0.7 (i.e., L* > 87), when the chromatic coordinates (*xyY*) of the sample are near the white point, ∆*E* values below 3.0 are obtained. In this zone (Y > 0.7), the gamut generated by each RGB representation encompasses several samples in the set, ensuring a highly reliable color interpretation. This is supported by the chromaticity diagrams in Figure 5b, where the coordinates calculated using Equation (S10) for six samples with varying *Y* values are displayed. Although all colors seem to be contained within the sRGB gamut, the volume defined by the *xyY* coordinates in Figure 5c reveals that those with the lowest *Y* values fall outside this volume, indicating that their properties cannot be accurately interpreted using RGB information.

The average values of the absolute color differences ${∆E}_{a}$, grouped by quadrants and limit values of L* (directly related to Y) are presented in Table 3. In all quadrants, particularly in Q3, ${∆E}_{a}$ values exceed 5.0 units, indicating a weak correlation between HSI and RGB data. For darker colors ($L^{*}$ < 50), these differences surpass 7.4 units in both RGB representations. In contrast, better reproducibility is observed when L* > 50, leading to a noticeable improvement in predicting ${∆E}_{a}$ < 5.0 for lighter colors (L* > 75). Although such color differences are accepted by many authors, these values remain still too high to establish a reliable correspondence between HSI and RGB [72,90,91,92].

Finally, Table 4 shows the analysis of the relative color difference ${∆E}_{r}$ (as defined in Section 2.2.5) for the HSI and RGB images, conducted using the Two One-Sided Test. This statistical method evaluates the equivalence of two approaches in reproducing similar results within a predefined tolerance.

The average values of the relative color differences for hyperspectral (${\bar{∆E}}_{r\;}HSI$) and RGB (${\bar{∆E}}_{r\;}RGB$) images decrease notably as the considered $L^{*}$ values increase. This is because the reference sample used to compute the relative differences in each case corresponds to the one with the highest lightness (first sample in the upper left corner- see Figure 2). A similar effect is observed when comparing the relative color differences obtained from HSI and RGB for each sample ($\bar{∆d}$), with values below 1.0, indicating an excellent reproduction of color variations for samples of $L^{*}$ > 75. Despite the high dispersion of the data in each interval ($\sigma_{d}$), the 95% confidence interval (IC 95%) comparing HSI and RGB indicates that most samples fall within a relative color difference below 3.0 units $L^{*}$ < 50, 2.0 units for $L^{*}$ > 50 and even lower to 1.0 units for $L^{*}$ > 75. Although the TOST results show data with statistically significant differences under the null hypothesis (*p* ≪ 0.05), with the size effect ranging from medium to small [93,94], the relative color differences found comparing HSI and RGB remain within the 3.0 units tolerance interval, supporting the practical equivalence of both methodologies for any lightness value.

These results indicate that, unlike absolute color differences, a relative color comparison does not require RGB to encompass the entire visible color space. Instead, knowing the coordinates of a specific reference point (sample reference), within the restricted space is sufficient to reliably quantify distances (color differences) to other points. In this way, the spectral bias inherent to RGB images is effectively canceled when computing distances between two points, making the resulting information equivalent to that obtained from hyperspectral data.

## 5. Conclusions

Six chromatic properties defined in the CIE-$L^{*}a^{*}b^{*}$ space were calculated and compared using HSI and RGB images in both sRGB and REC2020 representations. A Python algorithm was developed to process, individualize and analyze data from 700 Pantone^®^ fabric samples, aiming to establish a correlation between the color information obtained from each type of image. The results indicate that the accurate interpretation of the optical information from RGB images is strongly influenced by the lightness (L*) values due to the changes in the CIE-$L^{*}a^{*}b^{*}$ color space as it approaches the white reference. For samples with L* values greater than 50 (lighter colors), RPD values above 2.5 were found in four of the considered properties ($L^{*},{\;a}^{*},\;b^{*},\;{\;h}_{ab}$), indicating a good correlation between HSI and RGB data. The absolute color differences (${∆E}_{a}$) obtained from the direct comparison of HSI and RGB data exceeded 5.0 units for samples in the red-yellow-green quadrants (Q1 and Q2), rising to 9.0 for blues and purples (Q3 and Q4) in both sRGB and REC2020 representations. These discrepancies were attributed to the optical information captured by the RGB camera, which is strongly conditioned by the spectral response of the Bayer array filters (RGGB) in the cellphone, thereby limiting accuracy of the information in each channel. These differences decreased significantly in the relative color differences analysis (${∆E}_{r}$), which uses a reference sample for each HSI and RGB image. In this case, values below 3.0 units were achieved across the entire color space, reaching as low as 1.0 unit for light-colored samples ($L^{*}$ > 75). Statistical analysis using the Two One-Sided Test (TOST) indicate that, although HSI and RGB data show statistically significant differences under the null hypothesis (*p* ≪ 0.05), the relative differences between them remain within the 3.0 units tolerance interval commonly accepted in most academic industrial applications. Although RGB shows absolute limitations (with high ${∆E}_{a}$ values in red, blue, and purple tones), the use of relative color differences $({∆E}_{r}$) combined with statistical validation makes it a reliable and practical tool for comparative analyses. This approach results in a robust and validated protocol that can partially replace more expensive HSI systems in applications where absolute spectral reproduction is not essential, but relative color consistency is critical. Such capability is highly relevant for industries where reliable and cost-effective color assessment is required, including textiles and fashion (quality control of dyes and fabrics), printing and packaging (color matching and consistency across batches), food and beverages (monitoring freshness and product appearance), and digital imaging and display technologies (standardization of color rendering).

This work provides a solid first step toward RGB-based segmentation for fabric color analysis, offering valuable insights into its potential and limitations. Still, methodological extensions—such as incorporating multiple RGB devices with different Bayer filters, testing under varied illumination sources, and applying the approach to different material types—could significantly strengthen its robustness and broaden its applicability in industrial contexts.

## Figures and Tables

**Table 1:** Statistical analysis of the chromatic properties obtained using hyperspectral images separated by quadrants (Q) in terms of the $L^{*}$ (lightness) $a^{*},\;b^{*},\;{\;C}_{ab}^{*}$ (chroma), $h_{ab}$ (hue) and S (Saturation) for the 700 Pantone^®^ fabric samples (TCX).

Quadrant (N)		Color Properties CIE-L*a*b*					
		L*	a*	b*	$C_{ab}^{*}$	$h_{ab}$	S
**Q1** **(271)**	$\bar{x}$	59.11	24.58	18.56	34.00	39.20	45.78
	$x_{med}$	55.72	16.62	11.85	27.61	33.93	46.59
	$x_{min}$	27.02	0.02	0.08	1.93	0.22	3.63
	$x_{max}$	93.15	59.51	86.45	86.55	89.85	80.60
	$\bar{∆P}$	1.35	2.81	2.02	2.38	1.92	2.37
**Q2** **(168)**	$\bar{x}$	65.41	−15.44	21.35	29.54	129.06	38.86
	$x_{med}$	65.88	−13.18	15.39	26.67	129.85	38.48
	$x_{min}$	31.07	−50.69	0.31	3.50	90.14	5.20
	$x_{max}$	93.73	−0.02	81.20	81.35	179.39	68.36
	$\bar{∆P}$	1.96	1.10	1.45	1.35	2.06	2.85
**Q3** **(126)**	$\bar{x}$	56.70	−19.39	−19.73	31.27	226.67	48.52
	$x_{med}$	55.03	−18.42	−21.61	32.27	227.20	50.22
	$x_{min}$	30.60	−46.16	−36.13	3.60	180.04	11.69
	$x_{max}$	83.73	−0.27	−0.03	46.16	269.52	67.50
	$\bar{∆P}$	2.72	0.78	2.81	1.82	2.23	2.39
**Q4** **(135)**	$\bar{x}$	49.79	22.18	−12.28	28.08	325.64	47.53
	$x_{med}$	45.34	19.11	−12.38	29.31	333.50	52.19
	$x_{min}$	23.79	0.79	−34.89	2.44	271.74	6.23
	$x_{max}$	95.72	56.16	−0.10	56.20	359.23	76.09
	$\bar{∆P}$	1.96	2.51	1.86	2.75	2.20	2.55

N: number of samples; $\bar{x}$: average value; $x_{med}$: median; $x_{min}$: minimum; $x_{max}$: maximum; $\bar{∆P}$: average difference between HSI and Pantone^®^ CIE-L*a*b* properties.

**Table 2:** Accuracy of RGB images in reproducing the chromatic properties $L^{*}$ (lightness), $a^{*}$, $b^{*}$, $C_{ab}^{*}$(chroma), $h_{ab}$ (hue), and S (saturation), evaluated using the ratio of performance to deviation (RPD). The information has been separated by quadrant (Q) and $L^{*}$ intervals for sRGB and REC2020 representations.

Color Properties CIE-$L^{*}a^{*}b^{*}$		L*	a*	b*	$C_{ab}^{*}$	$h_{ab}$	S
		RPD					
**Q1**	sRGB	4.67	3.07	1.65	2.15	1.17	2.42
	REC 2020	4.41	3.32	1.64	2.13	0.85	2.50
**Q2**	sRGB	3.22	1.94	4.36	3.18	2.45	2.31
	REC 2020	3.23	1.83	3.57	2.80	2.31	2.17
**Q3**	sRGB	1.75	1.01	0.96	0.83	1.20	0.93
	REC 2020	1.60	1.46	0.87	0.84	1.41	0.87
**Q4**	sRGB	2.53	1.34	1.64	1.17	0.37	1.30
	REC 2020	2.41	1.44	1.75	1.20	0.74	1.28
$L^{*}$ **≤ 50**	sRGB	0.87	2.56	1.89	1.66	2.07	1.43
	REC 2020	0.83	2.77	2.08	1.74	2.29	1.44
$L^{*}$ **> 50**	sRGB	3.48	3.38	2.85	2.08	3.61	2.44
	REC 2020	3.28	3.91	2.57	1.97	3.14	2.39

**Table 3:** Average of the absolute (${∆E}_{a}$) color differences obtained by comparing HSI and RGB data in the sRGB and REC2020 representations. The information has been separated by quadrant (Q) and $L^{*}$ intervals for the 700 Pantone^®^ fabric samples (TCX). The standard deviation (σ) is included to show the precision of the results.

Samples	HSI vs. sRGB		HSI vs. REC 2020	
	${\bar{∆E}}_{a}$	±σ	${\bar{∆E}}_{a}$	±σ
**Q1**	5.68	2.03	5.57	2.03
**Q2**	5.57	2.51	5.71	2.42
**Q3**	9.69	2.33	10.52	2.28
**Q4**	6.80	1.45	6.74	1.66
$L^{*}$ **< 50**	7.54	1.73	7.43	1.59
$L^{*}$ **> 50**	5.72	2.67	5.68	2.51
$L^{*}$ **> 75**	3.91	1.77	4.03	1.86

**Table 4:** Summary of Two One-Sided Test analyses of the relative color difference (${∆E}_{r}$) calculated by comparing HSI and RGB data. The information has been separated by $L^{*}$ intervals for the 700 Pantone^®^ fabric samples (TCX).

Samples		$L^{*}$< 50	$L^{*}$> 50	$L^{*}$> 75
${\bar{∆E}}_{r}HSI$		27.72	16.15	6.73
${\bar{∆E}}_{r}RGB$	sRGB	31.69	17.89	7.60
	REC2020	32.13	18.55	7.82
$\bar{∆d}$	sRGB	2.48	1.44	0.29
	REC2020	2.66	1.95	0.37
$\sigma_{d}$	sRGB	3.27	2.04	0.73
	REC2020	3.41	2.11	0.84
CI95%	sRGB	[2.07, 2.89]	[1.24, 1.63]	[0.17, 0.41]
	REC2020	[2.23, 2.98]	[1.74, 1.83]	[0.23, 0.51]
***p* value** **(t paired)**	sRGB	<<0.05		
	REC2020			
**Effect size**	sRGB	0.75	0.70	0.40
	REC2020	0.77	0.72	0.44
**Conclusion**	sRGB	Relative color differences equivalent to HSI		
	REC2020			

${\bar{∆E}}_{r}$: average relative color difference; $\bar{∆d}$: average differences in HSI and RGB comparison; $\sigma_{d}$: standard deviation of $\bar{∆d}$; CI 95%: confidence interval of 95%.

## Data Availability

The original contributions presented in this study are included in the article. Further inquiries can be directed to the corresponding authors.

## Supplementary Materials

The following supporting information can be downloaded at https://www.mdpi.com/article/10.3390/jimaging12030116/s1: Figure S1: (a) CIE-*L***a***b** color space used to represent chromatic properties, with the L-axis associated with lightness ranging from 0 (black) to 100 (white). (b) CIE-*L***a***b** plane is divided into four quadrants based on a and b values, representing the chroma ($C_{ab}^{*}$) and hue ($h_{ab}$) for a given color; Figure S2: (a) Chromaticity diagram CIE-*xy* used for representation of visible color space (horseshoe) and gamut of sRGB (solid line) and REC2020 (dash line). (b) The Macadam ellipses allow to determine the region where a group of colors produce the same visual effect; Table S1: Digital data for the red (R), green (G) and blue (B) channels for the 16 samples of Figure 3 (main document), obtained from RGB camera. The colored squares have been generated by using the representations sRGB and REC2020 to highlight the differences (Diff) in color interpretation.

## Abbreviations

The following abbreviations are used in this manuscript:

HSI	Hyperspectral imaging
Q	Quadrant
RPD	Ratio to performance deviation
CIE	Commission Internationale d’Eclairage
L	Lightness
C_ab_	Chroma
h_ab_	Hue
TOST	Two One-Sided Test
SPD	Spectral Power Distribution
