# Supplementary material for: Assessing RGB Color Reliability via Simultaneous Comparison with Hyperspectral Data on Pantone® Fabrics
Source: J Imaging. 2026 Mar 10;12(3):116. doi: 10.3390/jimaging12030116 (PMC13028574; doi:10.3390/jimaging12030116)
Supplement: Supplementary file 1 [file jimaging-12-00116-s001.zip › jimaging-3797089-supplementary.pdf]

Section S1: CIE-$L^*a^*b^*$ color space

The CIE-$L^*a^*b^*$ color space, an international standard adopted by the Commission Internationale d'Eclaiage (CIE) in 1976, quantifies the sensitivity and uniformity of color changes perceived by human observers. It is represented as a three-dimensional vector system (Fig. S1a), where the coordinate L indicates the lightness of a color, ranging from black ($L^* = 0$) to white ($L^* = 100$). The a^* and b^* coordinates (-120, 120) determine the chroma (C^*_{ab}), hue (h_{ab}) and saturation (S) (see Eq. S1-S3) [1]. The a^* b^* plane of Fig. S1b is divided into four quadrants (Q) where Q1 ($+a^*$, $+b^*$) is the red-yellow region, Q2 ($-a^*$, $+b^*$) yellow-green, Q3 ($-a^*$, $-b^*$) green-blue, and Q4 ($+a^*$, $-b^*$) blue-purple [2–4].

$$C^*_{ab} = \sqrt{a^2 + b^2} \quad \text{(S1)}$$

$$h_{ab} = \tan^{-1}\left(\frac{b}{a}\right) \quad \text{(S2)}$$

$$S = \frac{C^*_{ab}}{\sqrt{C^{*2}_{ab} + L^{*2}}} * 100\% \quad \text{(S3)}$$

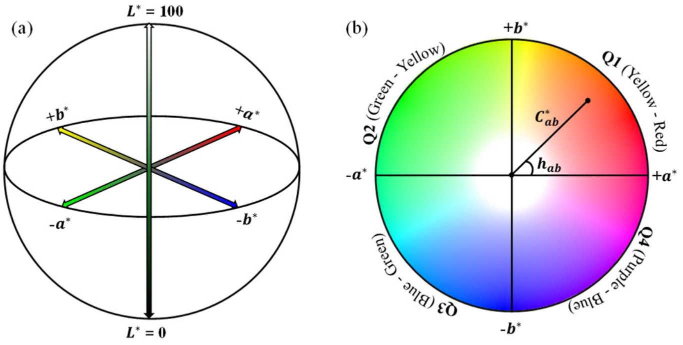

Figure S1. (a) CIE-$L^*a^*b^*$ color space used to represent chromatic properties, with the L^*-axis associated to lightness ranging from 0 (black) to 100 (white). (b) CIE-$L^*a^*b^*$ plane is divided into four quadrants based on a^* and b^* values, representing the chroma (C^*_{ab}) and hue (h_{ab}) for a given color.

The Euclidean distance in CIE-$L^*a^*b^*$ space was initially proposed as a measure of the similarity of two points (ΔE). This is calculated from the differences between their values of L^*, a^* and b^* (Eq. S4).

$$\Delta E_{76} = \sqrt{\Delta L^{*2} + \Delta a^{*2} + \Delta b^{*2}} \quad \text{(S4)}$$

Section S2: CIE- L* a* b* conversion

Tristimulus values CIE-XYZ

CIE-XYZ values are a mathematical representation of the human eye's response to light stimuli in the red (X), green (Y) and blue (Z) bands, corresponding to the sensitivity of the S, M and L cones in the retina. in the color's interpretation processes [5,6]. These values can be derived from the spectral fingerprint or the RGB values associates for a given sample.

Reflectance to XYZ

Reflectance spectra $r(\lambda)$ in the range from 380 to 780 nm provide the necessary data to accurately determine the chromatic properties of a surface. This calculation depends on the type of illumination $I(\lambda)$ and the color matching functions $\bar{x}(\lambda)$, $\bar{y}(\lambda)$ and $\bar{z}(\lambda)$ associated the optical capability of the observer [7–11].

The XYZ values are determined by weighted averages of these quantities using Eq. S5a-d.

$$X = K \int_{380nm}^{780nm} r(\lambda) I(\lambda)\, \bar{x}(\lambda) \mathrm{d}\lambda, \quad \text{(S5a)}$$

$$Y = K \int_{380nm}^{780nm} r(\lambda) I(\lambda)\, \bar{y}(\lambda) \mathrm{d}\lambda, \quad \text{(S5b)}$$

$$Z = K \int_{380nm}^{780nm} r(\lambda) I(\lambda)\, \bar{z}(\lambda) \mathrm{d}\lambda, \quad \text{(S5c)}$$

$$K = \frac{100}{\int_{380nm}^{780nm} I(\lambda)\, \bar{y}(\lambda) \mathrm{d}\lambda}, \quad \text{(S5d)}$$

Moreover, the CIE- L^* a^* b^* chromatic coordinates are calculated as follows:

$$L^* = 116 \left({}^{Y}\!/_{Y_n} \right)^{\frac{1}{3}} - 16, \quad \text{(S6)}$$

$$a^* = 500 \left[\left({}^{X}\!/_{X_n} \right)^{\frac{1}{3}} - \left({}^{Y}\!/_{Y_n} \right)^{\frac{1}{3}} \right], \quad \text{(S7)}$$

$$b^* = 200 \left[\left({}^{Y}\!/_{Y_n} \right)^{\frac{1}{3}} - \left({}^{Z}\!/_{Z_n} \right)^{\frac{1}{3}} \right], \quad \text{(S8)}$$

where X, Y, Z are the tristimulus associated to the sample, and X_n, Y_n, Z_n the corresponding values of the white reference [12,13].

RGB to XYZ

The RGB model is an additive color system that encodes the optical properties of an object using three components: red (R), green (G) and blue (B), emulating the operating principle of the human vision. In this system, white is represented by the maximum and equal values of each component (255, 255, 255), while black is by null values of these (0, 0, 0). Other colors are created by combinations of these three values [14,15].

Currently, different sizes (gamut) of visible space can be reproduced by RGB values, which differ primarily in the fidelity of colors (optical properties) projected on TV screens, computers and mobile devices. The most common color gamut used for consumer content today is the sRGB, created by Microsoft and Hewlett-Packard in 1996 to standardize color representation of web image and videos [16]. Recent color gamuts, such as REC2020, Adobe RGB or P65 have been developed to meet the requirements of high-definition displays, offering greater saturation (purity) [17]. REC2020 covers approximately 72% of the visible space, enhancing color fidelity compared to sRGB, which covers around 35%.

The transformation matrices that convert RGB values to CIE-XYZ color space, for an observer at 2° and a standard illuminant D65 in the sRGB and REC2020 representations, are shown in Eq. 9 a-b, respectively [18–21].

$$\begin{bmatrix} X \\ Y \\ Z \end{bmatrix} = \begin{bmatrix} 0.4124 & 0.3575 & 0.1804 \\ 0.2126 & 0.7151 & 0.0721 \\ 0.0193 & 0.1191 & 0.9502 \end{bmatrix} \begin{bmatrix} R_S \\ G_S \\ B_S \end{bmatrix} \quad \text{(S9a)}$$

$$\begin{bmatrix} X \\ Y \\ Z \end{bmatrix} = \begin{bmatrix} 0.6369 & 0.1446 & 0.1688 \\ 0.2627 & 0.6799 & 0.0593 \\ 0.0000 & 0.0280 & 0.0609 \end{bmatrix} \begin{bmatrix} R_R \\ G_R \\ B_R \end{bmatrix} \quad \text{(S9b)}$$

Chromaticity space CIE-xy

The tristimulus values are typically plotted on the CIE-xy chromaticity diagram using Eq. S10a-b. This is a colored solid whose contour represents the wavelengths of the visible region (380 to 700 nm). The interior of the horseshoe-shaped gamut illustrates the range of spectral combinations that can produce the colors perceivable by the human eye (see Fig. S2a). The "purple line" connecting the blue (380 nm) and red (700 nm) regions represent colors that exist only as mixtures of these wavelengths. The white reference point (D65 standard $x = 0.33$, $y = 0.33$) indicates an equal content of the three primaries (red, green, blue) and servers as reference for determining color saturation level. While colors are generally described trough three-dimensional systems (RGB, XYZ, L^* a^* b^*, xyY), the chromaticity diagram offers a simplified visualization of an object's chromatic properties.

$$x = \frac{X}{X+Y+Z}, \quad \text{(S10a)}$$

$$y = \frac{Y}{X+Y+Z}, \quad \text{(S10b)}$$

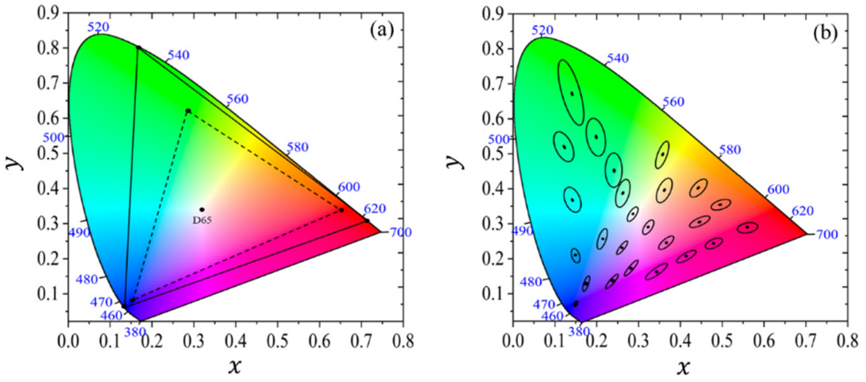

Figure S2. (a) Chromaticity diagram CIE-xy used for representation of visible color space (horseshoe) and gamut of sRGB (solid line) and REC2020 (dash line). (b) The Macadam ellipses allow to determine the region where a group of colors produce the same visual effect.

The chromaticity diagram in Fig. S2a illustrates the gamuts of sRGB and REC2020 representations. The REC2020 gamut encompasses a larger proportion of the visible color space (72%) compared to sRGB (35%), with notable differences in the blue and green regions [21]. In this study, the RGB images were analyzed in both color spaces before being converted to CIE-$L^*a^*b^*$ space to assess differences in color property calculations. Chromaticity space is also used to define regions around a reference point where color differences are barely perceptible to the human eye (just-noticeable difference-JND). Macadam et al. conducted matching tests in 25 chromatic regions, finding that equidistant points in the red, green or blue bands do not produce the same visual effect [22,23]. These regions, known as Macadam ellipses (Fig. S2b) are a key criterion for determining color differences in visual inspection processes.

Section S3: CIE-2000 color difference

Since the 1940s, several definitions of color differences have been developed for the CIE system. These range from simple relations, such as the Euclidean distance between two points in a 3D system (Eq. S4), to more complex empirical models that include adjustment parameters based on the color types evaluated. The most recent definition for color differences is CIE-2000 (ΔE), will be used in this work to compare the chromatic predictions using HSI and RGB images. Detailed descriptions of each parameter involved in Eq. S11 are available in Ref[24]

$$\Delta E = \left[\left(\frac{\Delta L'}{K_L S_L}\right)^2 + \left(\frac{\Delta C'}{K_C S_C}\right)^2 + \left(\frac{\Delta H'}{K_H S_H}\right)^2 + R_T\left(\frac{\Delta C'}{K_C S_C}\right)\left(\frac{\Delta H'}{K_H S_H}\right)\right]^{1/2}. \quad \text{(S11)}$$

Section S4: RGB values of representative Pantone TCX Samples

Table S1 displays the digital values associated to the chromatic information of the 16 samples of Fig. 3 (main document), obtained by using an RGB camera and represented in the gamut of sRGB and REC2020. In the Red-Yellow group (Q1), differences higher to 10% are found between both color representations in the three channels, particularly for dark samples. In the case of sample 3, the values are greater than 120 in the three cases, contributing equitably to the color representation generating between both gamut. These large or small differences between RGB values are well supported by the intensity level of the reflectance spectra in each wavelength band shown in Fig. 3a (main document). On the other hand, the variations between sRGB and REC2020 representations can be associated with the remarkable restriction and differences in the color space contained, thus affecting the capture and interpretation of the optical information, as shown in CIE-xy chromaticity diagram of Fig. S2a. Both representations mainly differ when R, G and/or B are lower than 120, which is corroborated by the findings of samples 5-8 (Q2) for channel B and samples 9-12 (Q3) for channel R. In the case of samples 14 and 16, despite having low values in two of the three channels, the discrepancy between sRGB and REC2020 is minimal. This is attributed to the fact that within the blue-purple region both representations exhibit similar gamut, resulting in equal color representation capabilities. Conversely, the most pronounced distinctions between the two representations are evident in the R channel for the darker samples within the green-blue spectrum. This occurs precisely in regions where there is minimal overlap between both gamut as well with the visible spectrum. Hence, the colors/samples in Q3 might be the ones generating the most significant disparities in the HSI and RGB color reproduction.

The most noticeable differences in the RGB values obtained from the two representations are observed when the optical characteristics of the sample are confined to a particular spectral region (Red, Green, Blue). This limitation directly impacts the accurate reproduction of the surface's color properties. In the case of high reflectance across the visible spectrum (associated with high L^* values), both RGB representations display a strong correspondence. Upon reviewing the color representations of the 16 samples (colored squares in Table S1) at first sight, there appears to be a trend where REC2020 tends to exhibit colors with a lighter appearance compared to sRGB. This discrepancy is attributed to the saturation level of the primaries defined in each case (red, blue green), which consequently impacting the representation of individual colors.

Table S1. Digital data for the red (R), green (G) and blue (B) channels for the 16 samples of Fig. 3 (main document), obtained from RGB camera. The colored squares have been generated by using the representations sRGB and REC2020 to highlight the differences (Diff) in color interpretation.

Samples		Color Representation		R			G			B		
		sRGB	REC 2020	sRGB	REC 2020	Diff (%)	sRGB	REC 2020	Diff (%)	sRGB	REC 2020	Diff (%)
Red – Yellow (Q1)	1			245.09	196.16	19.96	89.08	104.98	17.85	57.02	66.28	16.24
	2			168.00	129.89	22.68	30.03	48.73	62.27	49.08	51.12	4.16
	3			255.00	232.84	8.69	182.39	187.82	2.98	122.05	131.00	7.33
	4			249.57	198.49	20.47	86.10	103.08	19.72	30.29	46.10	52.20
Yellow – Green (Q2)	5			247.98	240.73	2.92	237.09	237.31	0.09	180.74	187.67	3.83
	6			223.09	204.95	8.13	185.73	187.45	0.93	51.96	77.30	48.77
	7			182.52	192.51	5.47	218.46	215.21	1.49	110.22	124.26	12.74
	8			176.91	174.91	1.13	177.81	177.23	0.33	129.68	135.36	4.38
Green – Blue (Q3)	9			220.55	227.35	3.08	240.56	239.42	0.47	214.94	217.84	1.35
	10			16.92	53.61	>100	90.47	87.25	3.56	68.92	70.69	2.57
	11			123.64	159.70	11.18	202.84	198.72	2.03	220.92	217.97	1.34
	12			0.13	50.16	>>100	104.50	99.96	4.34	107.24	105.81	1.33
Blue – Purple (Q4)	13			163.55	180.22	10.19	200.51	198.82	0.84	223.99	221.95	0.91
	14			41.46	61.13	10.22	77.93	76.78	1.48	116.57	113.39	2.73
	15			231.19	224.38	2.95	212.54	214.19	0.78	224.16	223.66	0.22
	16			45.35	43.91	3.18	36.21	37.50	3.56	70.08	67.56	3.60

References

1. León, K.; Mery, D.; Pedreschi, F.; León, J. Color Measurement in L*a*b* Units from RGB Digital Images. *Food Research International* 2006, *39*, doi:10.1016/j.foodres.2006.03.006.
2. Zhbanova, V.L. Research into Methods for Determining Colour Differences in the CIELAB Uniform Colour Space. *Light & Engineering* 2020, 53–59, doi:10.33383/2019-005.
3. Hill, B.; Roger, Th.; Vorhagen, F.W. Comparative Analysis of the Quantization of Color Spaces on the Basis of the CIELAB Color-Difference Formula. *ACM Trans Graph* 1997, *16*, 109–154, doi:10.1145/248210.248212.
4. Misue, K.; Kitajima, H. Design Tool of Color Schemes on the CIELAB Space. In Proceedings of the 2016 20th International Conference Information Visualisation (IV); IEEE, July 2016; pp. 33–38.
5. Wright, W.D. A Re-Determination of the Trichromatic Coefficients of the Spectral Colours. *Transactions of the Optical Society* 1929, *30*, 141–164, doi:10.1088/1475-4878/30/4/301.
6. J. Guild The Colorimetric Properties of the Spectrum. *Philosophical Transactions of the Royal Society of London. Series A, Containing Papers of a Mathematical or Physical Character* 1931, *230*, 149–187, doi:10.1098/rsta.1932.0005.
7. Wu, G.; Shen, X.; Liu, Z.; Yang, S.; Zhu, M. Reflectance Spectra Recovery from Tristimulus Values by Extraction of Color Feature Match. *Opt Quantum Electron* 2016, *48*, doi:10.1007/s11082-015-0325-9.
8. Cao, B.; Liao, N.; Li, Y.; Cheng, H. Improving Reflectance Reconstruction from Tristimulus Values by Adaptively Combining Colorimetric and Reflectance Similarities. *Optical Engineering* 2017, *56*, doi:10.1117/1.oe.56.5.053104.
9. Wu, G.; Qian, L.; Hu, G.; Li, X. Spectral Reflectance Recovery from Tristimulus Values under Multi-Illuminants. *Journal of Spectroscopy* 2019, *2019*, doi:10.1155/2019/3538265.
10. Xu, Y.; Zhang, C.; Gao, C.; Wang, Z.; Li, C. A Hybrid Adaptation Strategy for Reconstruction Reflectance Based on the given Tristimulus Values. *Color Res Appl* 2020, *45*, doi:10.1002/col.22495.
11. Bianco, S. Reflectance Spectra Recovery from Tristimulus Values by Adaptive Estimation with Metameric Shape Correction. *Journal of the Optical Society of America A* 2010, *27*, doi:10.1364/josaa.27.001868.
12. Autran, C. da S.; Gonçalez, J.C. Caracterização Colorimétrica Das Madeiras de Muirapiranga (Brosimum Rubescens-Taub.) e de Seringueira (Hevea Brasiliensis, Clone Tjir 16 Müll Arg.) Visando à Utilização Em Interiores. *Ciência Florestal* 2006, *16*, doi:10.5902/198050981926.
13. Bonfatti Júnior, E.A.; Lengowski, E.C. Colorimetria Aplicada à Ciência e Tecnologia Da Madeira. *Pesqui Florest Bras* 2018, *38*, doi:10.4336/2018.pfb.38e201601394.
14. Chernov, V.; Alander, J.; Bochko, V. Integer-Based Accurate Conversion between RGB and HSV Color Spaces. *Computers and Electrical Engineering* 2015, *46*, doi:10.1016/j.compeleceng.2015.08.005.
15. Ganesan, P.; Rajini, V.; Rajkumar, R.I. Segmentation and Edge Detection of Color Images Using CIELAB Color Space and Edge Detectors. In Proceedings of the International Conference on "Emerging Trends in Robotics and Communication Technologies", INTERACT-2010; 2010.
16. International Electrotechnical Commission Multimedia Systems and Equipment - Colour Measurement and Management - Part 2-1: Colour Management - Default RGB Colour Space - SRGB Available online: https://webstore.iec.ch/en/publication/6169 (accessed on 28 July 2024).
17. Süsstrunk, S.; Buckley, R.; Swen, S. Standard RGB Color Spaces. In Proceedings of the Final Program and Proceedings - IS and T/SID Color Imaging Conference; 1999.
18. López, F.; Valiente, J.M.; Baldrich, R.; Vanrell, M. Fast Surface Grading Using Color Statistics in the CIE Lab Space. In Proceedings of the Lecture Notes in Computer Science; 2005; Vol. 3523.
19. Hunt, R.W.G.; Pointer, M.R. *Measuring Colour: Fourth Edition*; 2011;
20. Froehlich, J.; Kunkel, T.; Atkins, R.; Pytlarz, J.; Daly, S.; Schilling, A.; Eberhardt, B. Encoding Color Difference Signals for High Dynamic Range and Wide Gamut Imagery. In Proceedings of the Final Program and Proceedings - IS and T/SID Color Imaging Conference; 2015; Vol. 2015-January.
21. Soneira, R.M. Display Color Gamuts: NTSC to Rec.2020. *Inf Disp (1975)* 2016, *32*, 26–31, doi:10.1002/j.2637-496X.2016.tb00920.x.
22. MacAdam, D.L. Visual Sensitivities to Color Differences in Daylight*. *J Opt Soc Am* 1942, *32*, 247, doi:10.1364/JOSA.32.000247.
23. Gravesen, J. The Metric of Colour Space. *Graph Models* 2015, *82*, doi:10.1016/j.gmod.2015.06.005.
24. Luo, M.R.; Rigg, B. BFD (l:C) Colour-difference Formula Part-1 Development of the Formula. *Journal of the Society of Dyers and Colourists* 1987, *103*, 86–94, doi:10.1111/j.1478-4408.1987.tb01099.x.
